# Treatment pattern and outcomes in *de novo* T790M-mutated non-small cell lung cancer

**DOI:** 10.3332/ecancer.2022.1385

**Published:** 2022-05-06

**Authors:** Goutam Santosh Panda, Vanita Noronha, Darshit Shah, George John, Anuradha Chougule, Vijay Patil, Rajiv Kumar, Nandini Menon, Ajay Singh, Pratik Chandrani, Abhishek Mahajan, Kumar Prabhash

**Affiliations:** Tata Memorial Hospital, Homi Bhabha National Institute (HBNI), Dr. E Borges Road, Parel, Mumbai 400012, India

**Keywords:** outcome, de novo T790M, non-small cell lung cancer, treatment pattern

## Abstract

**Introduction:**

Limited data exists for non-small cell lung cancer (NSCLC) patients harbouring *de novo* T790M mutation.

**Methods:**

NSCLC patients, with *de novo* T790M, who registered at our institute between 01/03/2015 and 31/12/2019, were considered for retrospective analysis of treatment pattern and clinical outcomes, i.e., progression-free survival (PFS) and overall survival (OS).

**Results:**

Of 1,542 epidermal growth factor receptor (*EGFR*)-mutated patients, 40 (2.59%) had *de novo* T790M. Most were male (27, 67.5%) and smokers (23, 57.5%). The commonest site of metastasis was the lungs (31, 77.5%), while 7 (17.5%) had central nervous system (CNS) involvement. Additional *EGFR* gene mutations and anaplastic lymphoma kinase (ALK) positivity were observed in 20 (50.0%) and 4 (10.0%) cases, respectively. The first-line systemic therapy and the number of patients receiving it were as follows: osimertinib by 14 (35.0%), first-generation *EGFR* tyrosine kinase inhibitors (TKIs) by 10 (25.0%), gefitinib + chemotherapy by 3 (7.5%), chemotherapy by 7 (17.5%) and gefitinib + bevacizumab by 2 (5%). One patient defaulted before starting any treatment. Hence, 39 were considered for survival analysis. The median PFS and OS for the entire cohort were 10.4 (95% CI = 7.6–19.7) months and 24.9 (95% CI = 15.7–NA) months, respectively. The median PFS for patients on osimertinib was 19.8 (95% CI = 11.6–28.0) months versus 8.8 (95% CI = 6.6–10.9) months for those on other systemic therapy. No CNS involvement, use of osimertinib or first-generation *EGFR* TKI plus chemotherapy or ALK inhibitor in ALK-positive cases prognosticated better PFS. When compared to other systemic therapies, osimertinib improved PFS in patients with or without additional *EGFR* mutations, although it was statistically significant for the former group only (*p* = 0.002).

**Conclusion:**

The incidence of *de novo* T790M is low. Osimertinib in frontline therapy provides promising outcomes.

## Introduction

Epidermal growth factor receptor (EGFR)-activating mutations are the most common driver mutations in non-small cell lung cancer (NSCLC). EGFR-activating mutations can be found in exons 18–21 of the *EGFR* gene. Exon 19 deletions and an exon 21 L858R point mutation are the most common mutations in patients with advanced NSCLC [1–3]. First- and second-generation *EGFR* tyrosine kinase inhibitors (EGFR-TKIs) outperform platinum-based treatment in NSCLC patients with activating EGFR mutations [4, 5]. Even better outcomes are observed in these patients with the use of third-generation *EGFR*-TKI or combining chemotherapy with first-generation TKI [6, 7]. Following TKI therapy, the T790M mutation accounts for 50% of the acquired resistance mutations. When receiving TKI treatment, this mutation is thought to arise under selection pressure. *De novo* T790M mutations occur at a different rate depending on the population examined and the mutation detection technology used. According to direct sequencing, this mutation can be detected in 0.4%–3% of all NSCLCs and 1%–8% of all EGFR-mutant NSCLCs. The T790M mutation causes threonine to be replaced with methionine at position 790. As a result, the T790M mutation’s affinity for ATP increases, outperforming the reversible inhibitors. Osimertinib, a third-generation EGFR-TKI, is approved for patients with T790M, but in second-line therapy. The T790M detection rate can be as high as 79% using more sensitive techniques, e.g., mutant-enriched polymerase chain reaction (PCR) and mass spectrometry [8–10]. However, barring a few trials with a small number of patients, no good data exist for patients with *de novo* T790M, which has been linked to inferior outcomes [8, 10–12]. Reversible TKIs show limited activity in these cases and usually cytotoxic chemotherapy is used to treat these patients [13]. Given the paucity of data regarding management and outcome of patients with *de novo* T790M, we undertook a retrospective study to analyse the treatment pattern and clinical outcomes of NSCLC patients with *de novo* T790 mutation.

## Methods

*De novo* T790M-mutated NSCLC patients who registered at our institute between 1/3/2015 and 31/12/2019 were considered for analysis. NSCLC patients harbouring additional driver mutations were also included. Prior history of malignancy was not an exclusion criterion. Approval from the Institutional Ethics Committee was taken and waiver for consent was obtained. No funding was used for this study.

The database maintained in the Department of Medical Oncology and Molecular Pathology was searched for *de novo* T790M-mutated patients. The World Health Organisation 2010 classification system was followed for making a histological diagnosis of NSCLC.

The primary objective of this study was to determine overall survival (OS). The secondary objectives were to study the pattern of care, determine the progression-free survival (PFS) and prognostic factors.

The QiaAmp formalin-fixed paraffin-embedded (FFPE) Tissue Kit was utilised for DNA extraction from six FFPE tissue sections of 14 mm each. The DNA was amplified for the *EGFR* exons 18–21 using a nested PCR method, followed by mutation analysis by TaqMan-based real-time PCR technique, as published earlier by our group [14].

The Response Evaluation Criteria in Solid Tumours 1.1 (RECIST 1.1) was employed for documenting response to therapy. The overall response rate (ORR) was calculated as the percentage of patients who achieved complete response (CR) or partial response (PR) on a particular therapy, while for the disease control rate (DCR), patients who attained stable disease, CR and PR were considered.

### Statistical analysis

Statistical analysis was carried out using SPSS (IBM SPSS Statistics for Windows, Version 25.0). The Kaplan–Meier method was used for estimating survival and the log-rank test was employed for comparison. Independent prognostic factors were determined by Cox’s multivariate analysis of significant prognostic factors on univariate analysis.

PFS was the period between diagnosis of metastatic carcinoma of the lungs and the first event, which might be progression (clinical and/or radiological), second malignancy or death. Patients who did not have an event or were lost to follow-up for more than 6 months were censored. OS was computed from the date of diagnosis to death from any cause or the last documented follow-up. The cut-off date for data collection was 1 December 2021.

## Results

### Baseline characteristics

**Figure 1.**
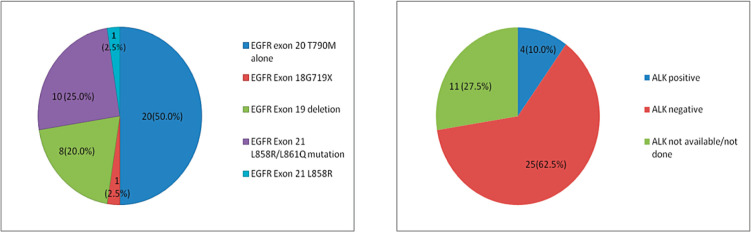
(a): Additional EGFR mutations in patients with *de novo* T790M. (b): Additional ALK rearrangement with *de novo* T790M.

We had 1,542 NSCLC patients who harboured *EGFR* mutations and were registered at our institute during the above-mentioned time period. *De novo* T790M mutations were detected in 40 of 1,542 patients (2.59%). There were 27 (67.5%) males and 13 (32.5%) females among the 40 patients, with 23 (57.5%) smokers and the rest were never smokers (Table 1). The median age at presentation was 54.5 years (range = 27–75). In four cases, granular cytoplasmic positivity for anaplastic lymphoma kinase 1 (ALK1) was observed by immunohistochemistry (IHC) and 20 (50%) patients also had other *EGFR* mutations in exons 18, 19 and 21, such as L858R/L861Q and G719X. Figure 1a and b shows the additional driver mutations found in these patients.

The most common site of metastasis was the lungs, followed by bone. Central nervous system (CNS) involvement at baseline was noted in seven (17.5%) patients. Brain imaging/cerebrospinal fluid (CSF) studies were considered for patients who had symptoms and/or signs of CNS involvement. The exceptions were NSCLC patients, with no evidence of metastasis on computed tomography (CT) thorax, for whom a whole-body fluorodeoxyglucose positron emission tomography and computed tomography (FDG PET/CT) was ordered to rule out metastasis. Prior history of malignancy (carcinoma of breast and ovary in one each) was noted in two patients, while one patient had synchronous non-secretory pituitary macro-adenoma.

### First-line (1L) treatment

Of these 40 patients, only one patient was non-metastatic at presentation and was initially treated with curative intent, i.e., surgical resection, followed by adjuvant chemotherapy. Later, this patient developed metastatic disease and was treated accordingly. The other 39 patients had metastatic disease at presentation. One patient defaulted before the start of any treatment at our institute and was not reachable. Hence, 39 patients were considered for survival analysis. Treatment for metastatic disease included first-generation TKI (erlotinib/gefitinib) alone in 10 (25.0%) patients, third-generation TKI (osimertinib) in 14 (35.0%) patients, gefitinib plus bevacizumab in 2 patients (5.0%), gefitinib plus chemotherapy in 3 (7.5%) patients, once-in-3-weeks pemetrexed and carboplatin doublet regimen in 6 out of 40 patients (15%), while etoposide and carboplatin doublet was given to 1 patient (Table 1). ALK inhibitor was advised for three out of four cases which were positive for ALK by IHC. No patient received combined targeted therapy, i.e., *EGFR* TKI plus ALK inhibitors.

### Overall response rate (ORR) and disease control rate (DCR)

ORR for the entire cohort was 48.7% and the DCR was 89.7%. The ORR and DCR for patients receiving osimertinib was 64.3% and 92.9%, respectively. The same for patients on systemic therapy other than osimertinib was 40% and 88%, respectively. This is presented in Table 2. The ORR and DCR with osimertinib for patients harbouring only T790M were 37.5% and 87.5%, respectively. For patients receiving systemic therapies other than osimertinib and having no additional *EGFR* mutation, the ORR and DCR were 41.7% and 83.3%, respectively.

### Treatment post-progression on 1L

Of all 39 patients analysed for survival, 26 patients had events for PFS (death = 2 patients; progression = 24 patients). Of these 26 patients, 18 patients received second-line therapy after progression on first-line systemic therapy, while the remaining 8 patients defaulted. The second-line treatment included osimertinib and chemotherapy in eight patients each, chemotherapy plus first-generation *EGFR*-TKI and ALK inhibitor in one patient each. Of all the patients experiencing events, repeat biopsy was carried out for only three patients and it could not be carried out for the rest of the patients due to various reasons such as poor ECOG PS post-progression, logistics for molecular testing and patient defaulting.

### Survival

The median follow-up in surviving patients was 34.9 (95% CI = 28.1–41.6) months. There were 26 (26/39, 66.7%) progressions and a total of 22 deaths, with no death attributable to the toxicity of systemic therapy.

**Figure 2.**
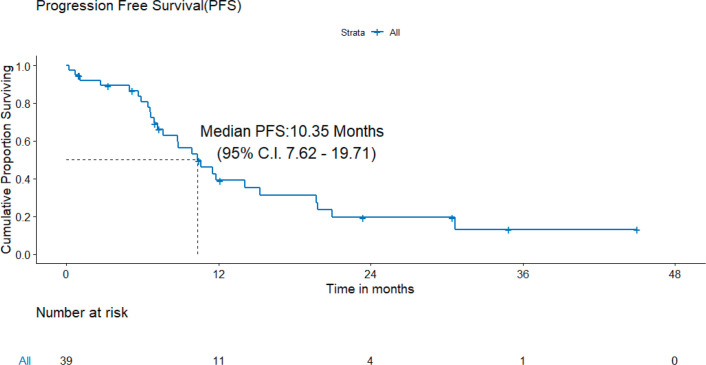
PFS of patients with *de novo* T790M.

A) PFS: At the time of analysis, 26 (26/39, 72.2%) patients experienced events for PFS. Only two patients failed in the brain. The median PFS was 10.4 (95% CI = 7.6–19.7) months (Figure 2). The projected 3-year PFS was 13.0%.

**Figure 3.**
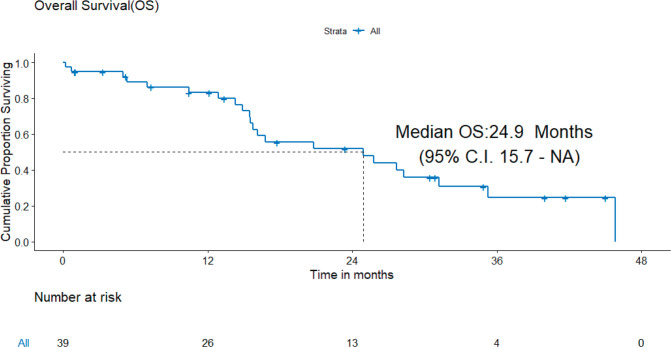
OS of patients with *de novo* T790M

B) OS: There were 22 deaths, none due to the toxicity of systemic therapy. The median OS was 24.9 (95% CI = 15.7–NA) months and the 3-year OS estimate was 24.6% (Figure 3).

### Patients with de novo T790M only

Osimertinib led to a median PFS and OS of 14.1 (95% CI = 4.6–23.6) months and 27.7 (95% CI = 0–56.7) months, respectively. Systemic therapies other than osimertinib led to median PFS and OS of 8.8 (95% CI = 4.5–13.0) and 14.9 (95% CI = 12.4–17.4) months in patients with *de novo* T790M-only *EGFR* mutation.

### Patients with additional EGFR mutations

On the contrary, patients on osimertinib who harboured additional *EGFR* mutations had median PFS and OS of 19.8 (95% CI = 19.7–20.0) months and 24.9 (95% CI = 17.6–32.2) months, respectively. The same for patients on other systemic therapies were 7.6 (95% CI = 4.6–10.6) months and 20.8 (95% CI = 13.2–28.4), respectively.

### Prognostic factors

**Figure 4.**
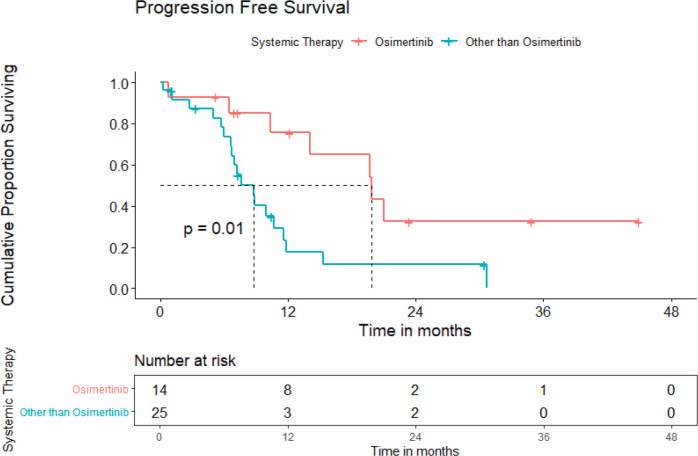
PFS for patients with *de novo* T790M using osimertinib versus other systemic therapies.

Entire cohort: In univariate analysis, no baseline CNS involvement (*p* = 0.04), using osimertinib (*p* = 0.01), using osimertinib/first-generation *EGFR* TKI plus chemotherapy/ALK inhibitor in ALK-positive NSCLC cases (*p* = 0.005) were predictors of superior PFS, while the only absence of CNS involvement was an indicator of better OS. The median PFS for patients on osimertinib was 19.8 (95% CI = 11.6–28.0) months versus 8.8 (95% CI = 6.6–10.9) months for those on other systemic therapy (Figure 4). The multivariate analysis could not be carried out due to fewer events. Poor PS at presentation tended to predict inferior OS (*p* = 0.051). Although osimertinib compared to other systemic therapies led to an improvement in PFS for both cohorts, i.e., patients with additional *EGFR* mutation and *de novo* T790M alone, it was statistically significant for patients with additional EGFR mutations only (*p* = 0.002).

## Discussion

The incidence of *EGFR* (exons 18–21) mutation in Indian ethnicity is 23%. Of these, *EGFR* exon 19 deletion and exon 21 mutation together constitute >90% [14]. The incidence of *de novo* T790M NSCLC varies from 1% to 38% and it depends on the detection method applied [2, 9, 12, 15]. We used real-time PCR with an assay sensitivity limit of detection of 1% for detecting *EGFR* mutations [14]. The incidence of T790M mutation at baseline was low in our cohort (2.59%). Two prior studies by our group also reported a low incidence of *de novo* T790M. In a study by Kate et al., only 10 (0.8%) of 1,260 patients harbouring an *EGFR* mutation had the *de novo* T790M mutation [16]. Similarly, the incidence of *de novo* T790M mutations reported by Noronha *et al* [17] was low (4/247; 1.6%) among patients with the EGFR exon 20-mutated lung cancer cases. However, a meta-analysis of 22 studies revealed the *de novo* T790M mutation rate to be in the range of 22%–80% [18]. Interestingly, we observed *EGFR* T790M at a 2.6% frequency, more in line with another study from China [19]. We suggest future experiments with highly sensitive NGS or droplet digital PCR (ddPCR) for a better assessment of T790M mutation rate in the Indian cohort. Most of the patients were non-smokers as seen with other *EGFR*-sensitising mutations [6, 7, 20]. The incidence of baseline CNS involvement in our study was 17.5%. This corroborates with our previous studies [7, 21] in which the incidence was 13.9% and 18%, respectively. The reported incidence of CNS involvement in *EGFR*-positive NSCLC ranges from 20% to 55% [22–26]. A co-existing *EGFR* mutation was noted in approximately half of the patients (19/40, 47.5%) in our study, similar to other studies [27, 28].

The T790M is known to be insensitive to first-generation TKIs. Hence, for patients with the T790M mutation, osimertinib is recommended, but only in the second or subsequent lines of treatment. The clinical outcomes for these patients have been studied in few retrospective studies with a limited sample size. ORR for patients on osimertinib in our cohort was 64.3%. The response rate with osimertinib was 85.7% in the AURA trial, in which 6 out of 7 patients with T790M mutation had a response to osimertinib [29]. An objective response rate of 80% in osimertinib arm and 76% in the standard *EGFR* TKI arm was observed in a phase-3 randomised trial of patients with untreated NSCLC harbouring exon 19 deletion or L858R mutation in *EGFR* gene [6]. Similarly, osimertinib in second line for NSCLC patients with T790M conferred an objective response rate of 71% [30]. The median PFS of patients with *de novo* T790M was superior statistically when treated with osimertinib and so was OS, although not statistically significant. However, in our cohort, patients with *de novo* T790M alone had inferior response rates (ORR and DCR) and survival outcomes (PFS and OS) compared to those having additional *EGFR* mutations. However, these worse outcomes were not statistically significant. The inferior outcomes in patients with *de novo* T790M alone could be due to the absence of other *EGFR* driver mutations, especially sensitising ones, small sample size, retrospective the nature of study and a real-world scenario, which has its own intricacies.

The outcome of patients harbouring *de novo* T790M was poor with a median PFS of 10 months, while the same for patients with sensitising *EGFR* mutations was 16 months on gefitinib+chemotherapy [7]. Our median PFS of 10 months in these patients with *de novo* T790M is likewise comparable to the result reported by other investigators with a limited sample size [9, 12, 31]. The median PFS was slightly higher (23.1 months (95% CI = 14.1–NE)) for the recently published phase-IIa study comprising 22 patients, with all receiving osimertinib [32] compared to the median PFS of 19.8 months in our cohort of patients receiving osimertinib. The possible explanations for higher PFS are small sample size, T790M coexistent in low allelic frequency with exon 19 deletion or L858R mutation and separate ethnicity from our cohort. However, the response rate and disease control rate were similar (77.3% and 86.4%, respectively). In our study, although the outcome with gefitinib+chemotherapy was comparable to osimertinib, a small number of patients (only three) received the former treatment. Hence, a firm conclusion cannot be drawn on the efficacy of gefitinib plus chemotherapy for these patients. Moreover, osimertinib compared to other systemic therapies led to superior PFS in patients with or without additional *EGFR* mutations, although the improvement in PFS was statistically significant for those with additional EGFR mutations.

### Limitation

The limitation of this study includes all the limitations of a retrospective study. Also, we did not analyse the patients’ allelic frequency as a prognostic factor, which may have an impact on the outcome. Although carried out in a limited number of patients, its clinical relevance cannot be overlooked due to the rarity of such cases and the lack of any standard guidelines. We report the outcome of such patients with relatively good follow-up.

## Conclusion

The incidence of *de novo* T790 mutations in NSCLC is low. Osimertinib can be considered as frontline therapy for these patients with a promising outcome. Future prospective studies are needed for better understanding of disease biology, treatment and prognosis of patients with this rare mutation.

## Abbreviations

*EGFR* = Epidermal growth factor receptor

TKI = Tyrosine kinase inhibitor

NSCLC = Non-small cell lung cancer

CNS = Central nervous system

ALK = Anaplastic lymphoma kinase

RECIST 1.1 = Response Evaluation Criteria in Solid Tumours 1.1

PFS = Progression-free survival

OS = Overall survival

FDG PET/CT = Fluorodeoxyglucose positron emission tomography and computed tomography

## Conflicts of interest/competing interests

None.

## Funding

No financial assistance was utilised for this study.

## Ethical approval

Approval from institutional ethics committee (IEC) was taken and waiver of consent was obtained from IEC.

## Figures and Tables

**Table 1.** Baseline parameters of patients with *de novo* T790M mutation.

Patients’ characteristics	*N* = 40 (%)
**Age** in years, Median (Range)	54.5 (27–75)
**Gender** Male Female	27 (67.5%) 13 (22.5%)
**ECOG PS** 0–1 2 >2	19 (47.5%) 14 (35.0%) 7 (17.5%)
**Comorbidity** Yes Hypertension Diabetes mellitus Others (hypothyroidism, ulcerative colitis and ischemic heart disease) No	19 (47.5%) 14 (35%) 8 (20%) 4 (10%) 21 (52.5%)
**Histology** Adenocarcinoma Adenosquamous carcinoma Combined large cell neuroendocrine carcinoma and squamous carcinoma.	38 (95.0%) 1 (2.5%) 1 (2.5%)
**Smoking status** Never smoker Ever smoker Current smoker	17 (42.5%) 23 (57.5%) 0 (0%)
**Baseline AJCC stage** I II III IV	0 (0%) 1 (2.5%) 0 (0%) 39 (97.5%)
**Initial sites of metastasis** Lungs (Parenchymal/(pleural deposits and effusion)) Bone Liver CNS Adrenal gland Non-regional lymph node Others	31(77.5%) (19(47.5%)/17 (42.5%)) 16 (40%) 6 (15%) 7 (17.5%) 4 (10%) 4 (10%) 4 (10%)
First-line treatment for metastatic disease EGFR TKI alone a. Gefitinib b. Erlotinib c. Osimertinib Chemotherapy only a. Pemetrexed and carboplatin b. Carboplatin and etoposide Combination of chemotherapy and TKI ALK inhibitor Defaulted before any treatment Other (gefitinib plus bevacizumab)	24 (60%) 6 (15%) 4 (10%) 14 (35%) 7 (17.5%) 6 (15%) 1 (2.5%) 3 (7.5%) 3 (7.5%) 1 (2.5%) 2 (5%)

Abbreviations: ECOG PS = Eastern Cooperative Oncology Group performance status; EGFR = epidermal growth factor receptor; EGFR-TKI = EGFR tyrosine kinase inhibitor; TKI = tyrosine kinase inhibitor; CNS = central nervous system; N = number

**Table 2.** ORR and DCR with various treatments for patients with *de novo* T790M.

Treatment (*n* = 39)	ORR (%)	DCR (%)
Osimertinib (*n* = 14)	64.3%	92.9%
Systemic therapies other than osimertinib (*n* = 25) 1. First-generation *EGFR* TKI (*n* = 10) 2.Chemotherapy (*n* = 7) 3.Chemotherapy + EGFR TKI (*n* = 3) 4. ALK inhibitor (*n* = 3) 5. Other (Gefitinib plus Bevacizumab) (*n* = 2)	40.0% 20.0% 42.9% 33.3% 100.0% 50.0%	88.0% 90.0% 85.7% 100.0% 100.0% 50.0%

**Abbreviations:** *EGFR* TKI = Epidermal growth factor receptor tyrosine kinase inhibitor; ALK = Anaplastic lymphoma kinase
